# Supplementary material for: Microbiome of Hyalomma dromedarii (Ixodida: Ixodidae) Ticks: Variation in Community Structure with Regard to Sex and Host Habitat
Source: Insects. 2024 Dec 27;16(1):11. doi: 10.3390/insects16010011 (PMC11766007; doi:10.3390/insects16010011)
Supplement: Supplementary file 1 [file insects-16-00011-s001.zip › insects-3310963-supplementary.pdf]

Supplementary Table SA. Read quality detail (Results of Assembly (by FLASH).

Read Quality by Sample

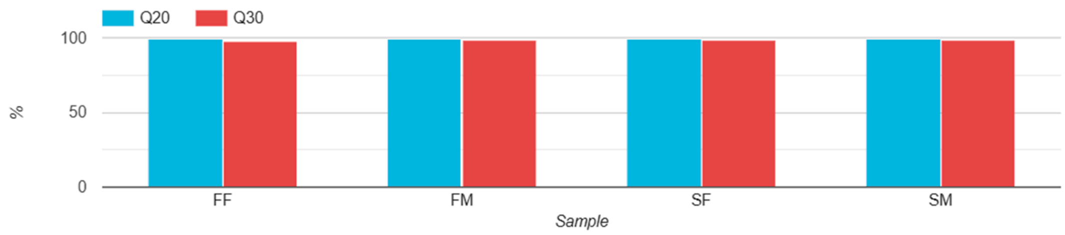

SampleName	Total Bases	Read Count	N(%)	GC(%)	Q20(%)	Q30(%)
FF	32,780,382	72,220	0.0001	53.42	99.43	97.88
FM	47,477,429	102,291	0.0001	53.45	99.53	98.28
SF	36,589,995	79,221	0.0001	50.73	99.46	98.04
SM	39,996,443	87,412	0.0001	51.31	99.53	98.24

* Total Bases : The total number of bases in reads indentified
* Read Count : The total number of sequence reads
* N(%) : The N percentage in sequence reads
* GC(%) : The GC percentage in sequence reads
* Q20(%) : The percentage of bases in which the phred score is above 20
* Q30(%) : The percentage of bases in which the phred score is above 30

Supplementary Table SB. Read count detail (Summary - Preprocessing and Clustering (by CD-HIT-OTU).

OTU Picking Method: de novo

- Results of Clustering (cutoff : 97%)

No.	SampleName	Read Count
1	FF	41,658
2	FM	36,063
3	SF	33,946
4	SM	39,501

- Results of Preprocessing

Sample Count	4
Read Count	151,168
Gamma-diversity	237
**Counts/sample summary**	
Min	33,946.0
Max	41,658.0
Median	37,782.0
Mean	37,792.0
**Filtered Read Count**	
Ambiguous	176
Wrong prefix or primers	13,960
(Sequence of prefix or primer)	CCTACGGG[ACGT]GGC[AT]GCAG
Low-Quality	209
Chimera	10,873
Other	164,758

* Sample Count : The total number of sample
* Read Count : The total number of sequence reads
* Gamma-diversity represents the diversity across an entire landscape. (alpha + beta diversity)
* Alpha-diversity corresponds to species diversity in sites/habitats at a local scale
* Beta-diversity comprises species diversity among sites/habitats
* Min : Minimum number of sequence per samples
* Max : Maximum number of sequence per samples
* Median : The number separating the higher half of a data samples
* Mean : The average number of the sequence of samples
* Ambiguous : Filtered seqs with ambiguous base calls
* Low-Quality : Filtered seqs with low-quality bases (Quality score offset 33)
* Chimera : Filtered seqs with chimeric reads
* Denoising : Filtered seqs with all other noise

Supplementary Table S1. Microbial phyla (presence in %) detected in *H. dromedarii* ticks from two habitats in the UAE.

Sr. No.	Phylum	FF	FM	SF	SM
1.	Actinomycetota	35.83	4.23	4.12	6.94
2.	Bacillota	37.22	3.74	19.51	47.35
3.	Bacteroidota	0.18	0.00	1.55	5.61
4.	Balneolota	0.00	0.00	0.00	0.12
5.	Cyanobacteriota	0.04	0.00	0.00	0.90
6.	Deinococcota	0.00	0.00	0.01	0.26
7.	Fusobacteriota	0.02	0.00	0.65	2.31
8.	Gemmatimonadota	0.00	0.00	0.00	0.03
9.	Myxococcota	0.00	0.00	0.00	0.02
10.	Pseudomonadota	26.58	92.02	74.17	36.11
11.	Verrucomicrobiota	0.13	0.00	0.00	0.01

Supplementary Table S2. Microbial classes (presence in %) detected in *H. dromedarii* ticks from two habitats in the UAE.

Sr. No.	Class	FF	FM	SF	SM
1.	Actinomycetes	35.83	4.23	4.07	6.68
2.	Nitriliruptoria	0.00	0.00	0.00	0.18
3.	Bacilli	20.58	1.94	13.87	31.97
4.	Clostridia	1.28	0.19	1.63	2.71
5.	Erysipelotrichia	0.58	0.05	0.35	2.56
6.	Tissierellia	14.79	1.57	3.63	10.08
7.	Bacteroidia	0.18	0.00	1.31	3.58
8.	Flavobacteriia	0.01	0.00	0.24	2.03
9.	Balneolia	0.00	0.00	0.00	0.12
10.	Cyanophyceae	0.04	0.00	0.00	0.90
11.	Deinococci	0.00	0.00	0.01	0.26
12.	Fusobacteriia	0.02	0.00	0.65	2.31
13.	Alphaproteobacteria	0.01	0.00	0.22	0.81
14.	Betaproteobacteria	0.87	0.12	1.27	1.61

15.	Gammaproteobacteria	25.70	91.90	72.68	33.69
16.	Spartobacteria	0.00	0.00	0.00	0.01
17.	Verrucomicrobiae	0.13	0.00	0.00	0.00

Supplementary Table S3. Microbial orders (presence in %) detected in *H. dromedarii* ticks from two habitats in the UAE.

Sr. No.	Order	FF	FM	SF	SM
1.	Actinomycetales	1.10	0.24	0.52	0.83
2.	Micrococcales	2.83	0.12	1.52	0.91
3.	Mycobacteriales	31.74	3.87	2.02	4.93
4.	Pseudonocardiales	0.15	0.00	0.00	0.00
5.	Euzebyales	0.00	0.00	0.00	0.18
6.	Bacillales	12.47	1.04	3.75	25.35
7.	Lactobacillales	8.11	0.89	10.12	6.61
8.	Eubacteriales	1.28	0.19	1.63	2.71
9.	Erysipelotrichales	0.58	0.05	0.35	2.56
10.	Tissierellales	14.79	1.57	3.28	8.79
11.	Bacteroidales	0.10	0.00	1.31	3.57
12.	Flavobacteriales	0.01	0.00	0.24	2.03
13.	Balneolales	0.00	0.00	0.00	0.12
14.	Nodosilineales	0.04	0.00	0.00	0.90
15.	Deinococcales	0.00	0.00	0.01	0.20
16.	Fusobacteriales	0.02	0.00	0.65	2.31
17.	Hyphomicrobiales	0.01	0.00	0.09	0.47
18.	Rhodobacterales	0.00	0.00	0.11	0.29
19.	Burkholderiales	0.02	0.00	0.06	0.95
20.	Neisseriales	0.84	0.12	1.20	0.66
21.	Enterobacterales	0.02	89.83	0.11	1.35
22.	Moraxellales	22.12	1.23	1.84	2.45
23.	Pasteurellales	1.76	0.22	0.06	0.02
24.	Pseudomonadales	0.00	0.03	0.10	0.27
25.	Thiotrichales	1.76	0.60	70.55	29.56
26.	Verrucomicrobiales	0.13	0.00	0.00	0.00

Supplementary Table S4. Microbial families (presence in %) detected in *H. dromedarii* ticks from two habitats in the UAE.

Sr. No.	Family	FF	FM	SF	SM
1.	Actinomycetaceae	1.10	0.24	0.52	0.83
2.	Kytococcaceae	0.17	0.00	0.00	0.05
3.	Microbacteriaceae	0.00	0.00	0.12	0.06
4.	Micrococcaceae	2.58	0.11	1.39	0.80

5.	Corynebacteriaceae	31.74	3.87	2.02	4.89
6.	Pseudonocardiaceae	0.15	0.00	0.00	0.00
7.	Euzebyaceae	0.00	0.00	0.00	0.18
8.	Bacillaceae	1.42	0.02	0.26	2.33
9.	Staphylococcaceae	11.05	1.02	3.42	23.02
10.	Aerococcaceae	8.00	0.82	0.90	0.66
11.	Carnobacteriaceae	0.01	0.00	0.00	0.15
12.	Lactobacillaceae	0.00	0.00	1.25	0.43
13.	Streptococcaceae	0.10	0.07	7.91	5.37
14.	Clostridiaceae	0.08	0.01	0.09	0.27
15.	Eubacteriaceae	0.00	0.00	0.00	0.15
16.	Lachnospiraceae	0.13	0.00	0.27	0.45
17.	Oscillospiraceae	0.18	0.00	0.11	0.13
18.	Peptostreptococcaceae	0.64	0.16	0.94	1.57
19.	Erysipelotrichaceae	0.00	0.00	0.14	1.79
20.	Turicibacteraceae	0.58	0.05	0.21	0.77
21.	Peptoniphilaceae	14.79	1.57	3.28	8.79
22.	Porphyromonadaceae	0.06	0.00	0.32	1.02
23.	Prevotellaceae	0.03	0.00	0.77	2.55
24.	Rikenellaceae	0.00	0.00	0.23	0.00
25.	Weeksellaceae	0.00	0.00	0.21	1.97
26.	Balneolaceae	0.00	0.00	0.00	0.12
27.	Nodosilineaceae	0.04	0.00	0.00	0.90
28.	Deinococcaceae	0.00	0.00	0.01	0.20
29.	Fusobacteriaceae	0.02	0.00	0.62	2.30
30.	Methylobacteriaceae	0.00	0.00	0.07	0.14
31.	Nitrobacteraceae	0.00	0.00	0.00	0.21
32.	Rhizobiaceae	0.00	0.00	0.01	0.12
33.	Paracoccaceae	0.00	0.00	0.11	0.29
34.	Sphingomonadaceae	0.00	0.00	0.00	0.04
35.	Comamonadaceae	0.02	0.00	0.06	0.26
36.	Oxalobacteraceae	0.00	0.00	0.01	0.17
37.	Neisseriaceae	0.84	0.12	1.20	0.66
38.	Enterobacteriaceae	0.01	0.00	0.01	0.39
39.	Morganellaceae	0.01	89.83	0.10	0.96
40.	Moraxellaceae	22.12	1.23	1.84	2.45
41.	Pasteurellaceae	1.76	0.22	0.06	0.02
42.	Pseudomonadaceae	0.00	0.03	0.10	0.27
43.	Francisellaceae	1.76	0.60	70.55	29.56
44.	Akkermansiaceae	0.13	0.00	0.00	0.00

Supplementary Table S5. Microbial genera (presence in %) detected in *H. dromedarii* ticks from two habitats in the UAE.

Sr. No.	Genus	FF	FM	SF	SM

1.	*Gleimia*	0.00	0.00	0.30	0.57
2.	*Trueperella*	1.10	0.24	0.22	0.26
3.	*Kytococcus*	0.17	0.00	0.00	0.05
4.	*Clavibacter*	0.00	0.00	0.09	0.05
5.	*Rothia*	2.58	0.11	1.39	0.73
6.	*Corynebacterium*	31.74	3.87	2.02	4.89
7.	*Amycolatopsis*	0.15	0.00	0.00	0.00
8.	*Euzebya*	0.00	0.00	0.00	0.18
9.	*Alkalicoccobacillus*	0.00	0.00	0.00	0.11
10.	*Alteribacillus*	0.01	0.00	0.00	0.14
11.	*Bacillus*	0.23	0.00	0.00	0.38
12.	*Halalkalibacter*	0.17	0.00	0.00	0.35
13.	*Lysinibacillus*	0.06	0.01	0.11	0.44
14.	*Metabacillus*	0.00	0.00	0.00	0.10
15.	*Niallia*	0.20	0.01	0.04	0.36
16.	*Oceanobacillus*	0.15	0.00	0.00	0.14
17.	*Paraliobacillus*	0.10	0.00	0.00	0.00
18.	*Peribacillus*	0.44	0.01	0.06	0.10
19.	*Aliicoccus*	0.00	0.00	0.39	0.71
20.	*Salinicoccus*	0.04	0.00	1.33	0.42
21.	*Staphylococcus*	11.01	1.02	1.69	21.89
22.	*Abiotrophia*	2.74	0.10	0.01	0.00
23.	*Globicatella*	5.25	0.72	0.85	0.66
24.	*Atopostipes*	0.00	0.00	0.00	0.14
25.	*Weissella*	0.00	0.00	1.22	0.43
26.	*Streptococcus*	0.10	0.07	7.91	5.37
27.	*Fenollaria*	0.00	0.00	0.16	0.00
28.	*Neofamilia*	0.19	0.02	0.00	0.01
29.	*Alkaliphilus*	0.00	0.00	0.00	0.09
30.	*Clostridium*	0.08	0.01	0.09	0.18
31.	*Eubacterium*	0.00	0.00	0.00	0.15
32.	*Anaerobutyricum*	0.00	0.00	0.00	0.28
33.	*Blautia*	0.12	0.00	0.07	0.00
34.	*Herbinix*	0.00	0.00	0.00	0.10
35.	*Faecalibacterium*	0.18	0.00	0.00	0.00
36.	*Paeniclostridium*	0.30	0.08	0.42	0.74
37.	*Peptostreptococcus*	0.16	0.02	0.14	0.26
38.	*Romboutsia*	0.18	0.05	0.38	0.58
39.	*Holdemania*	0.00	0.00	0.14	1.79
40.	*Turicibacter*	0.58	0.05	0.21	0.77
41.	*Ezakiella*	0.00	0.00	0.28	0.86
42.	*Kallipyga*	0.00	0.00	0.07	0.43
43.	*Anaerococcus*	0.65	0.06	1.03	3.06

44.	*Finegoldia*	0.00	0.00	0.10	0.13
45.	*Helcococcus*	2.18	0.15	0.66	3.72
46.	*Parvimonas*	0.00	0.00	0.16	0.56
47.	*Peptoniphilus*	11.91	1.34	1.32	1.32
48.	*Porphyromonas*	0.06	0.00	0.32	1.02
49.	*Hallella*	0.00	0.00	0.08	0.34
50.	*Hoylesella*	0.00	0.00	0.01	0.39
51.	*Prevotella*	0.03	0.00	0.68	1.77
52.	*Alistipes*	0.00	0.00	0.23	0.00
53.	*Chryseobacterium*	0.00	0.00	0.10	0.45
54.	*Epilithonimonas*	0.00	0.00	0.00	1.25
55.	*Ornithobacterium*	0.00	0.00	0.11	0.27
56.	*Gracilimonas*	0.00	0.00	0.00	0.12
57.	*Marileptolyngbya*	0.04	0.00	0.00	0.90
58.	*Deinococcus*	0.00	0.00	0.01	0.20
59.	*Fusobacterium*	0.02	0.00	0.62	2.30
60.	*Methylorubrum*	0.00	0.00	0.00	0.13
61.	*Bradyrhizobium*	0.00	0.00	0.00	0.21
62.	*Agrobacterium*	0.00	0.00	0.01	0.10
63.	*Paracoccus*	0.00	0.00	0.11	0.23
64.	*Aquabacterium*	0.00	0.00	0.00	0.51
65.	*Ottowia*	0.00	0.00	0.06	0.26
66.	*Janthinobacterium*	0.00	0.00	0.00	0.12
67.	*Neisseria*	0.00	0.00	0.04	0.12
68.	*Uruburuella*	0.84	0.12	1.14	0.54
69.	*Escherichia*	0.01	0.00	0.01	0.39
70.	*Proteus*	0.01	89.83	0.10	0.96
71.	*Acinetobacter*	0.00	0.00	0.00	0.38
72.	*Moraxella*	21.97	1.21	1.84	1.87
73.	*Psychrobacter*	0.15	0.02	0.00	0.19
74.	*Mannheimia*	1.76	0.22	0.02	0.00
75.	*Pseudomonas*	0.00	0.03	0.10	0.27
76.	*Francisella*	1.76	0.60	70.55	29.56
77.	*Akkermansia*	0.13	0.00	0.00	0.00

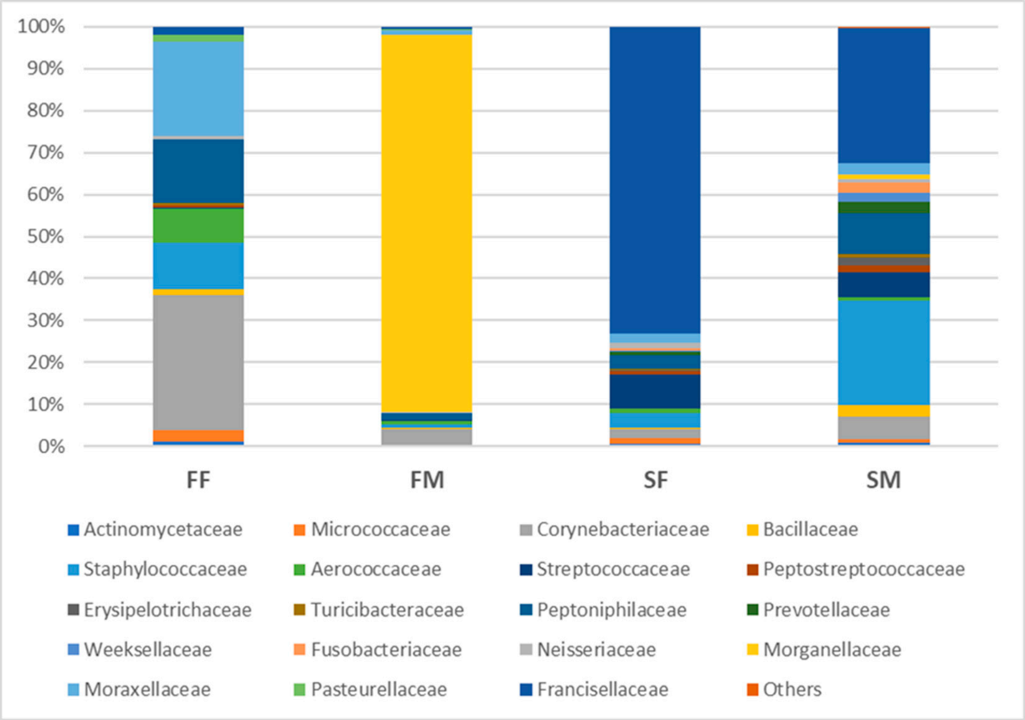

Supplementary Figure S1: Microbial families detected in *H. dromedarii* ticks collected from different habitats, farm and slaughterhouse, respectively. Abbreviations: FF, Farm Female, FM, Farm Male, SF, Slaughterhouse Female, SM, Slaughterhouse Male.

Supplementary Figure 2. Operational Taxonomic Unit (OTU).

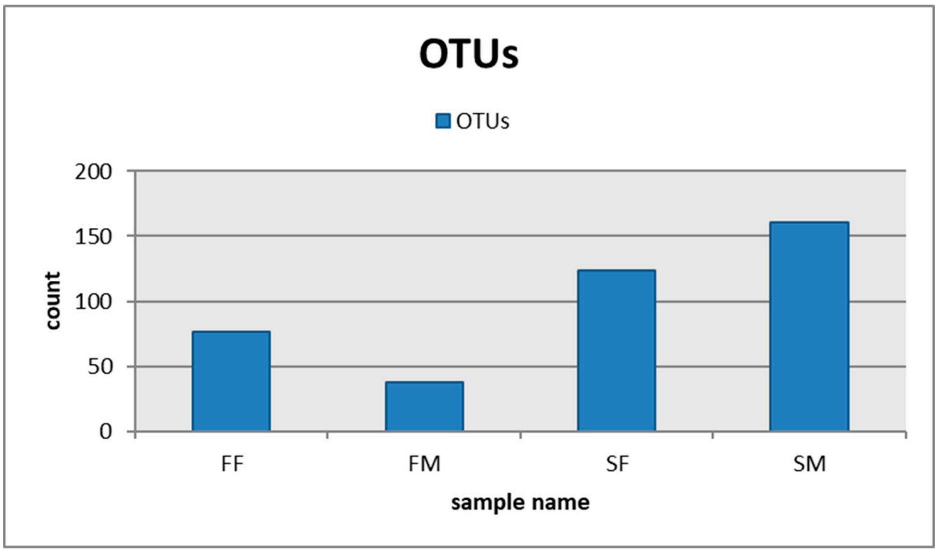

Supplementary Table S6: Community richness and diversity

Community richness & diversity

SampleName	OTUs	Chao1	Shannon	Gini-Simpson	Good's Coverage
FF	76	76	3.8200565	0.892237693	1
FM	38	38.2	0.866866	0.192186666	0.999944541
SF	124	124	2.4745766	0.498933647	0.999970541
SM	161	161	4.4587336	0.859312385	1

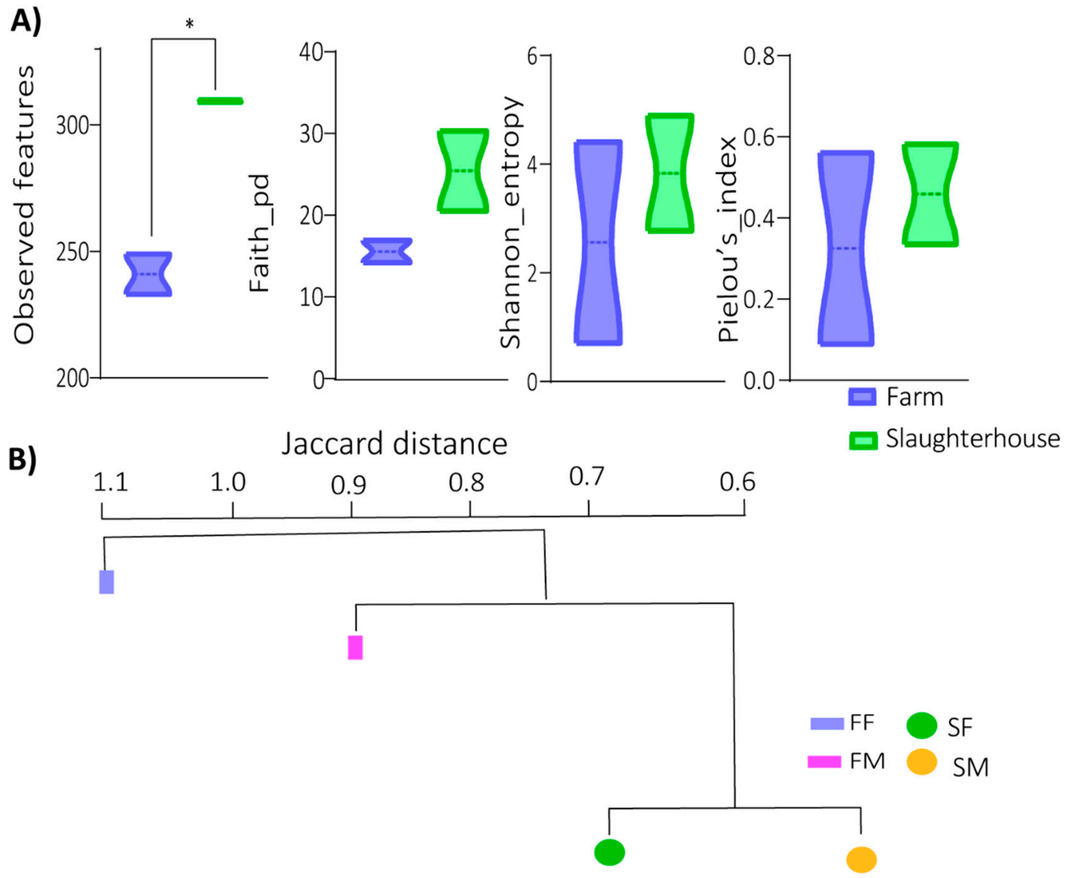

Supplementary Figure S3: Comparison of microbial diversity across the habitat. (A) Comparison of alpha diversity with observed features, Faith_pd, Shanon entropy and Pielou's evenness index for farm (purple) and slaughterhouse (green) (Kruskal Wallis $p > 0.05$) B) Jaccard clustering of samples (FF, FM, SF and SM). The samples are represented by circles and the groups by colors (legend).

Supplementary Table S7: Topological features of the taxonomic networks representing different on the tick habitat.

Networks features	Farm	Slaughterhouse
Nodes	113	219
Edges	758	2116
Positives	318 (41,95%)	1042 (49,24%)
Negatives	440 (58,04%)	1074 (50,76%)
Modularity	-2,675	-102,12
Number of Communities	51	44
Network diameter	2	3
Average Degree	13,41	19,32
Weighted degree	-2,114	-0,267
Clustering coefficient (Triangles method)	0,43	0.67
Triangles number	4646	29716
Connectivity	1	1

Supplementary Table S8: Microbiome studies in different *Hyalomma* tick species in the MENA region.

Tick species	Origin	Developmental stage/Sex	Tissue	Location	Target gene	Approach	Year	Prevalent genus	Reference
Hyalomma dromedarii	Field collected ticks	Adults	Whole tick	Saudi Arabia	V3–V4 region of 16S rRNA gene	Illumina MiSeq	2019	*Francisella* was the most abundant with average abundance of 94.37%.	[1]
Hyalomma dromedarii	Field collected ticks	Adults	Whole tick	Palestine	V3–V4 region of 16S rRNA gene and Shot gun metagenomic sequencing	Illumina and NextSeq 500	2019	*Francisella*	[2]

Hyalomma dromedarii	Field collected ticks	Adults	Whole tick	Saudi Arabia	V3–V4 region of<br>16S rRNA gene	Illumina MiSeq	2020	*Pseudomonas* (45.37%).	[3]
Hyalomma dromedarii	Field collected ticks	Adults female	Whole tick	Al-Ain (UAE)	V3–V4 region of<br>16S rRNA gene	Illumina MiSeq	2020	*Acinetobacter* (75.66%) in 2010 samples<br>*Francisella* (99%) in 2019 samples	[4]
Hyalomma anatolicum	Field collected ticks	Adult female and eggs	Whole tick/eggs	Iran	V3 region of<br>16S rRNA gene	Illumina MiSeq	2021	*Francisella* had a relative abundance of 96.8% in ticks from cattle	[5]
Hyalomma dromedarii	Field collected ticks	Adults female	Whole tick	Al-Ain (UAE)	V3–V4 region of<br>16S rRNA gene	Illumina MiSeq	2022	*Francisella* (79.4%)	[6]
Hyalomma anatolicum	Field collected ticks	Adults male	Whole tick	Al-Ain (UAE)	V3–V4 region of<br>16S rRNA gene	Illumina MiSeq	2022	*Staphylococcus* and *Corynebacterium* (57.6% and 41.5%, respectively) from ticks collected from sheep. *Francisella* had a relative abundance of 72% in ticks collected from goats, followed by *Proteus* (57.9%) from cows.	[7]

Reference

1. Elbir, H.; Almathen, F.; Alhumam, N.A. A Glimpse of the Bacteriome of Hyalomma dromedarii Ticks Infesting Camels Reveals Human Helicobacter Pylori Pathogen. *J Infect Dev Ctries* **2019**, *13*, 1001–1012, doi:10.3855/jidc.11604.

2. Ravi, A.; Ereqat, S.; Al-jawabreh, A.; Abdeen, Z.; Abu, O.; Id, H.H.; Id, M.J.P.; Nasereddin, A. Metagenomic Profiling of Ticks: Identification of Novel Rickettsial Genomes and Detection of Tick-Borne Canine Parvovirus. *PLoS Negl Trop Dis* **2019**, *13*, 1–19.

3. Alreshidi, M.M.; Veettil, V.N.; Noumi, E.; Campo, R. Del; Snoussi, M. Description of Microbial Diversity Associated with Ticks Hyalomma dromedarii (Acari: Ixodidae) Isolated from Camels in Hail Region (Saudi Arabia) Using Massive Sequencing of 16S RDNA. *Bioinformation* **2020**, *16*, 602–610, doi:10.6026/97320630016602.

4. Perveen, N.; Muzaffar, S.B.; Vijayan, R.; Al-Deeb, M.A. Microbial Communities Associated with the Camel Tick, Hyalomma dromedarii: 16S RRNA Gene-Based Analysis. *Sci Rep* **2020**, *10*, 1–11, doi:10.1038/s41598-020-74116-7.

5. Choubdar, N.; Karimian, F.; Koosha, M.; Oshaghi, M.A. An Integrated Overview of the Bacterial Flora Composition of Hyalomma anatolicum, the Main Vector of CCHF. *PLoS Negl Trop Dis* **2021**, *15*, 1–15, doi:10.1371/journal.pntd.0009480.

6. Perveen, N.; Muzaffar, S.B.; Vijayan, R.; Al-Deeb, M.A. Assessing Temporal Changes in Microbial Communities in Hyalomma dromedarii Collected from Camels in the UAE Using High-Throughput Sequencing. *Front Vet Sci* **2022**, *9*, 1–12, doi:10.3389/fvets.2022.861233.

7. Perveen, N.; Muzaffar, S.B.; Vijayan, R.; Al-Deeb, M.A. Microbial Composition in Hyalomma anatolicum Collected from Livestock in the United Arab Emirates Using Next-Generation Sequencing. *Parasit Vectors* **2022**, *15*, 1–9, doi:10.1186/s13071-021-05144-z.
